# Left atrium mass in a patient with breast cancer: a case report

**DOI:** 10.1186/s13256-016-1079-0

**Published:** 2016-10-19

**Authors:** Yu Sugawara, Ryuji Okamura, Shigeki Taniguchi

**Affiliations:** 1 Department of Internal Medicine, Yamatotakada Municipal Hospital, 1-1, Isono-Cho, Yamatotakada-Shi, Nara-Ken Japan; 2 Department of Surgery, Yamatotakada Municipal Hospital, 1-1, Isono-Cho, Yamatotakada-Shi, Nara-Ken Japan; 3 Department of Cardiovascular Surgery, Nara Medical University, 840, Shijo-Cho, Kashihara-Shi, Nara-Ken Japan

**Keywords:** Apixaban, Thrombus, Left atrial mass

## Abstract

**Background:**

Left atrial thrombi have traditionally been treated with heparin and warfarin, and many physicians have limited experience with direct oral anticoagulants such as apixaban. Furthermore, the efficacy of apixaban for the treatment of left atrial thrombi has not been established. We experienced a case of left atrial thrombus formation before breast cancer surgery, which was resolved by apixaban.

**Case presentation:**

Computed tomography for a 74-year-old Japanese woman with breast cancer incidentally revealed a left atrial mass with a root before the breast surgery. The mass was surgically removed and determined to be a thrombus. Before the breast surgery, transthoracic echocardiography was performed again, and the left atrial thrombus had recurred within only 14 days. It resolved after administration of apixaban.

**Conclusions:**

A left atrial thrombus might recur within a very short time. Apixaban might be an alternative to warfarin in patients with breast cancer and left atrial thrombus.

## Background

Left atrial thrombus is one of the differential diagnoses of a left atrial mass and is usually observed in the left atrial appendage. Historically, left atrial thrombi have been treated with heparin and warfarin. Recently, direct oral anticoagulants (DOACs) have been developed, and the superiority of DOACs to warfarin in preventing stroke and reducing bleeding has been demonstrated [1]. However, the efficacy of a DOAC such as apixaban for the treatment of left atrial thrombus has not been established. We experienced a case of a left atrial mass in a patient with breast cancer. The mass was a thrombus that recurred in only 14 days and then resolved completely after approximately 2 months of apixaban administration.

## Case presentation

**Fig. 1:**
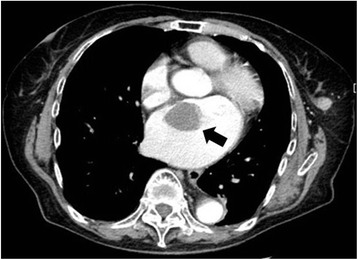
A 30 × 30-mm mass in the left atrium on contrast-enhanced computed tomography of the chest (black arrow)

A 74-year-old Japanese woman (height 136 cm; weight 40 kg) without systemic disease presented to our hospital with a palpable 15-mm mass in her left breast that was identified as stage I breast cancer. Before surgery for removal of the mass, a contrast-enhanced pulmonary computed tomography (CT) was performed and showed a mass approximately 30 × 30 mm in her left atrium (Fig. 1).

**Fig. 2:**
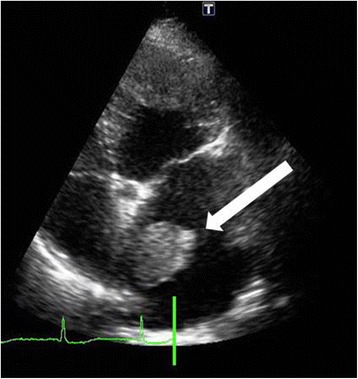
Transthoracic echocardiography showing a 33 × 30-mm mass in the left atrium (white arrow)

Based on transthoracic echocardiography (TTE), her left ventricle ejection fraction was 65.3 % and her left atrial diameter was 42 mm; the mass was 33 × 30 mm, it had a root, it was located on the surface of the septal wall, and was oscillating (Fig. 2). An electrocardiogram showed paroxysmal atrial fibrillation. Her chest radiography was normal. Her CHA_2_DS_2_-VASc score was 2, CHADS_2_ score was 0, and HAS-BLED score was 1. Laboratory investigations revealed the following: hemoglobin level, 12.8 g/dL; serum creatinine level, 0.67 mg/dL; estimated glomerular filtration rate, 64.6 mL/minute/1.73 m^2^; brain natriuretic peptide, 199.2 pg/dL; international normalized ratio (INR), 1.04; activated partial thromboplastin time (APTT), 32.5 seconds; protein C, 113 %; protein S, 75 %; antinuclear antigen, 40; and lupus anticoagulant, 1.4 seconds. Her levels of cancer antigen 15-3 and NCC-ST 439 were 23.8 U/mL and 1.3 U/mL, respectively.

**Fig. 3:**
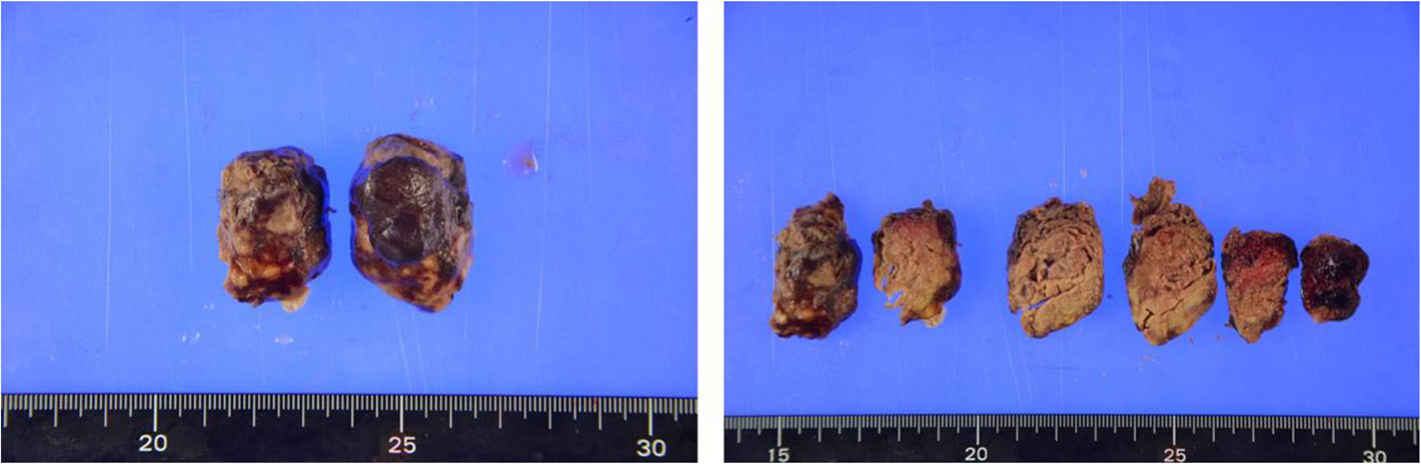
Left atrial mass pathology showing the mass was composed of a thrombus, without tumor tissue

Cardiac surgery was performed 16 days after her first visit to remove the left atrial mass. On pathologic examination, the mass consisted of a thrombus, without tumor cells (Fig. 3). After removal of the left atrial mass, heparin was initiated, and breast surgery was planned.

**Fig. 4:**
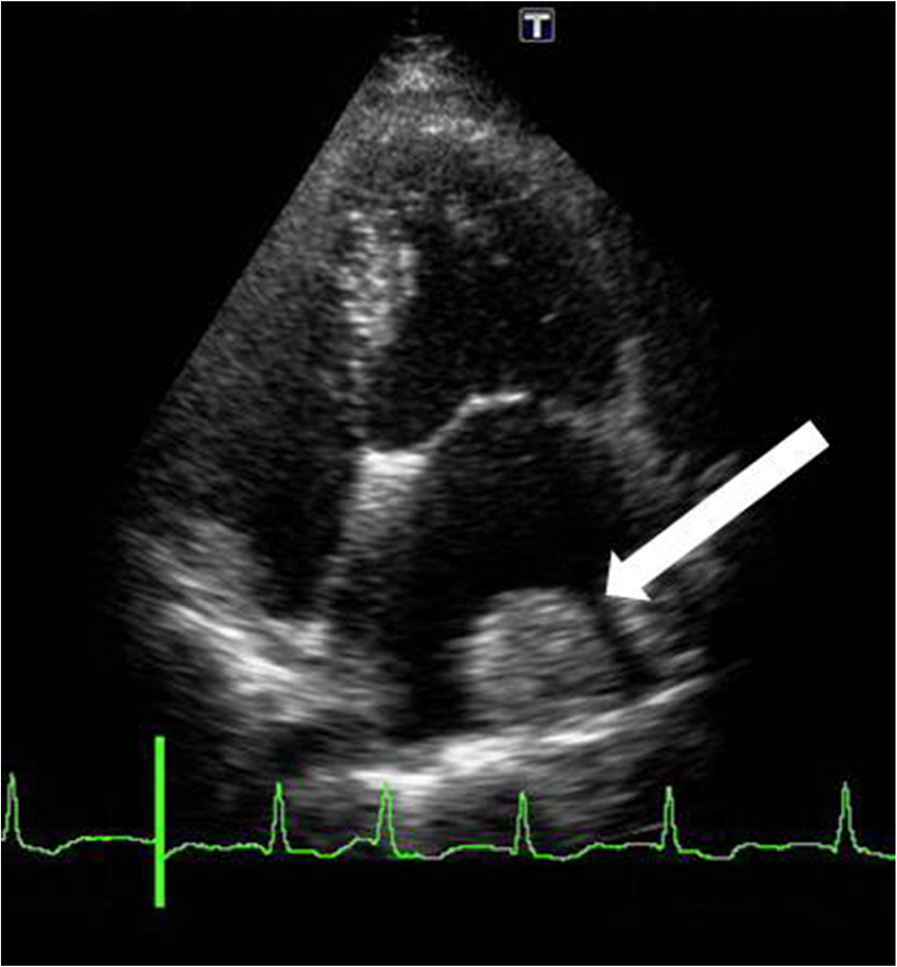
Fourteen days after cardiac surgery, a thrombus had formed over the lateral left atrium wall (white arrow)

**Fig. 5:**
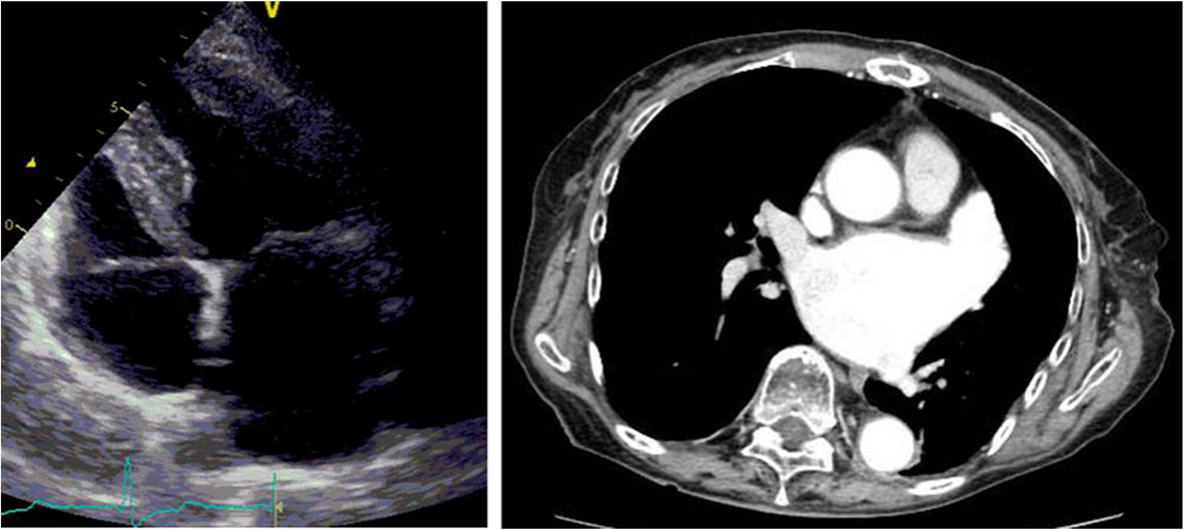
The cardiac thrombus disappeared on transthoracic echocardiography and contrast-enhanced computed tomography of the chest post-apixaban treatment

Fourteen days after her cardiac surgery, TTE was performed, showing a mass formation in the lateral wall of her left atrium (Fig. 4). The mass was 38 × 31 mm and was potentially a thrombus. Therefore, her breast operation was postponed and 1 mg/day of letrozole was administered to prevent progression of the breast cancer. The left atrial thrombus was treated with heparin and warfarin. Because her INR level fluctuated, rivaroxaban was administered. However, owing to gastrointestinal upset, she did not take rivaroxaban; therefore, 5 mg of apixaban twice a day was prescribed. Approximately 2 months later, TTE and contrast-enhanced pulmonary CT showed resolution of the left atrial thrombus (Fig. 5). After complete resolution of the left atrial thrombus, her breast surgery was performed. During the anticoagulation therapy, thromboembolism and lethal hemorrhage did not occur.

## Discussion

This case highlighted three important clinical issues. First, apixaban effectively resolved the left atrial thrombus. DOACs such as apixaban have recently been developed and are superior to warfarin for preventing stroke and reducing bleeding [1]. Although there have been some case reports of resolution of a left atrial thrombus with apixaban [2–4], the mechanisms of thrombus resolution are not fully understood. One possible mechanism is that antithrombin is not required for the antithrombotic activity of apixaban, which is a selective factor Xa inhibitor. It inhibits both free and clot-bound factor Xa as well as prothrombinase activity. It indirectly inhibits platelet aggregation and decreases thrombin generation and fibrin clot development [5, 6]. In addition, the fibrinolytic system plays a role in thrombus resolution. Because of the limited experience with DOACs for left atrial thrombus resolution, additional cases and further investigation are needed to establish the efficacy of apixaban for this purpose.

Second, the cardiac thrombus relapsed in a very short time after removal. Our patient had paroxysmal atrial fibrillation and cancer. Malignancy has a significant role in a thrombus, and venous and arterial thromboembolisms are common complications for patients with cancer [7]. In a normal coagulation-fibrinolysis system, there is a natural balance between activation and inhibition of procoagulants and anticoagulants. Cancer cells can alter this balance through the production of cancer-related procoagulants such as tissue factor and cancer procoagulant [8]. Tissue factor is a transmembrane glycoprotein and the primer of the physiological coagulation cascade. Cancer procoagulant, a cysteine protease, is a direct activator of factor X and is found in malignant tissues. Cancer procoagulant, in the presence of factor V, enhances thrombin production [9]. Tumor-specific activation of factor X might be an important step in the blood coagulation cascade in patients with cancer. In our case, we used a direct Xa inhibitor (apixaban), which resulted in a favorable outcome.

Finally, differential diagnoses of intracardiac mass are a benign tumor, malignant tumor, metastatic tumor, and thrombus. Atrial myxoma is the most common primary intracardiac tumor in adults; two thirds of myxomas arise in the left atrium [10]. However, cardiac thrombi typically occur in the left atrial appendage. In the present case, the left atrial mass had a root and oscillated, which are typical characteristics of myxomas and atypical characteristics of a left atrial thrombus.

## Conclusion

Apixaban is a favorable alternative therapy for patients with a cardiac thrombus.

## Abbreviations

CT: Computed tomography
DOAC: Direct oral anticoagulant
INR: International normalized ratio
TTE: Transthoracic echocardiography
